# Crystallographic and spectroscopic snapshots reveal a dehydrogenase in action

**DOI:** 10.1038/ncomms6935

**Published:** 2015-01-07

**Authors:** Lu Huo, Ian Davis, Fange Liu, Babak Andi, Shingo Esaki, Hiroaki Iwaki, Yoshie Hasegawa, Allen M. Orville, Aimin Liu

**Affiliations:** 1 Department of Chemistry, Georgia State University, Atlanta, Georgia 30303, USA; 2 Molecular Basis of Disease Area of Focus Program, Georgia State University, Atlanta, Georgia 30303, USA; 3 Photon Sciences Directorate, Brookhaven National Laboratory, Upton, New York 11973, USA; 4 Department of Life Science and Biotechnology and ORDIST, Kansai University, Suita, Osaka 564-8680, Japan; 5 Biosciences Department, Brookhaven National Laboratory, Upton, New York 11973, USA

## Abstract

Aldehydes are ubiquitous intermediates in metabolic pathways and their innate reactivity can often make them quite unstable. There are several aldehydic intermediates in the metabolic pathway for tryptophan degradation that can decay into neuroactive compounds that have been associated with numerous neurological diseases. An enzyme of this pathway, 2-aminomuconate-6-semialdehyde dehydrogenase, is responsible for ‘disarming’ the final aldehydic intermediate. Here we show the crystal structures of a bacterial analogue enzyme in five catalytically relevant forms: resting state, one binary and two ternary complexes, and a covalent, thioacyl intermediate. We also report the crystal structures of a tetrahedral, thiohemiacetal intermediate, a thioacyl intermediate and an NAD^+^-bound complex from an active site mutant. These covalent intermediates are characterized by single-crystal and solution-state electronic absorption spectroscopy. The crystal structures reveal that the substrate undergoes an *E*/*Z* isomerization at the enzyme active site before an *sp*^3^-to-*sp*^2^ transition during enzyme-mediated oxidation.

## Abstract (web summary)

Aldehydes are common intermediates in enzymatic pathways, but their high reactivity can make them difficult to observe. Here, the authors study the mechanism of aldehyde deactivation in a dehydrogenase, showing a key *E/Z* isomerization and observing a thiohemiacetal intermediate by crystal structure analysis.

## Graphical abstract

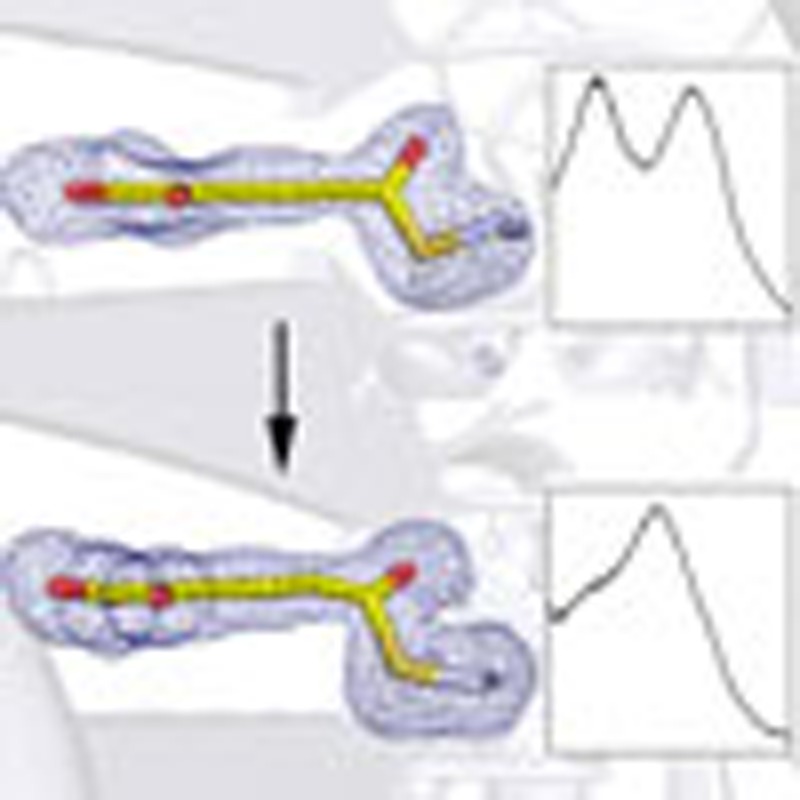

The dominant route of tryptophan catabolism, the kynurenine pathway, has recently garnered increased attention given its apparent association with numerous inflammatory and neurological conditions, for example, gastrointestinal disorders, depression, Parkinson’s disease, Alzheimer’s disease, Huntington’s disease and AIDS dementia complex^1^,^2^,^3^,^4^,^5^,^6^. Though the precise mechanism by which the kynurenine pathway influences these diseases has not yet been fully elucidated, it has been determined that several metabolites of this pathway are neuroactive. Notably, the concentration of quinolinic acid, a non-enzymatically derived decay product of an intermediate of the kynurenine pathway used for NAD^+^ biosynthesis, is elevated over 20-fold in patients’ cerebrospinal fluid with AIDS dementia complex, aseptic meningitis, opportunistic infections or neoplasms^7^, and more than 300-fold in the brain of human immunodeficiency virus-infected patients^8^. This NAD^+^ precursor has also been shown to be an agonist of *N*-methyl-D-aspartate receptors, and an increase of its concentration may lead to over-excitation and death of neuronal cells^9^,^10^.

The apparent medical potential of the kynurenine pathway warrants detailed study and characterization of its component enzymes and their regulation. One enzyme in particular, 2-aminomuconate-6-semialdehyde dehydrogenase (AMSDH), is responsible for oxidizing the unstable metabolic intermediate 2-aminomuconate-6-semialdehyde (2-AMS) to 2-aminomuconate (2-AM) (Fig. 1a). On the basis of sequence alignment, AMSDH is a member of the hydroxymuconic-semialdehyde dehydrogenase (HMSDH) family under the aldehyde dehydrogenase (ALDH) superfamily^11^. ALDHs are prevalent in both prokaryotic and eukaryotic organisms and are responsible for oxidizing aldehydes to their corresponding carboxylic acids. They use NAD(P)^+^ as a hydride acceptor to harvest energy from their primary substrate and generate NAD(P)H, which provides the major reducing power to maintain cellular redox balance^12^,^13^. In addition to being commonly occurring metabolic intermediates, aldehydes are reactive electrophiles, making many of them toxic. Enzymes of the ALDH superfamily are typically promiscuous with regards to their substrates; however, in recent years, this superfamily has had several new members identified with greater substrate fidelity, especially when the substrate is identified as a semialdehyde^14^.

The putative native substrate of AMSDH, 2-AMS, is a proposed metabolic intermediate in both the 2-nitrobenzoic acid degradation pathway of *Pseudomonas fluorescens* KU-7 (ref. 15) and the kynurenine pathway for L-tryptophan catabolism in mammals^9^,^10^,^16^. In the presence of NAD^+^ and AMSDH, 2-AMS is oxidized to 2-AM (Fig. 1a); however, it can also spontaneously decay to picolinic acid and water with a half-life of 35 s at neutral pH^17^. Due to its instability, 2-AMS has not yet been isolated, leaving its identity as the substrate of AMSDH an inference based on decay products and further metabolic reactions. There are several reasons for the poor understanding of this pathway: it is complex with many branches, some of the intermediates are unstable and difficult to characterize, and several enzymes of the pathway, including AMSDH, are not well understood. Hence, the structure of AMSDH will help to address questions such as what contributes to substrate specificity for the semialdehyde dehydrogenase and how 2-AMS is bound and activated during catalysis.

In the present study, we have cloned AMSDH from *Pseudomonas fluorescens*, generated an *E. coli* overexpression system and purified the target protein for molecular study. We also constructed several mutant expression systems to characterize the role of specific active site residues. Enzymatic assays were performed for all forms of the enzyme, and crystal structures were solved for the wild type and one mutant. We were able to capture several catalytic intermediates *in crystallo* by soaking protein crystals in mother liquor containing either the primary organic substrate or a substrate analogue and discovered that in addition to dehydrogenation, the substrate undergoes isomerization at the active site.

## Results

### Catalytic activity of wild-type AMSDH

**Figure 1:**
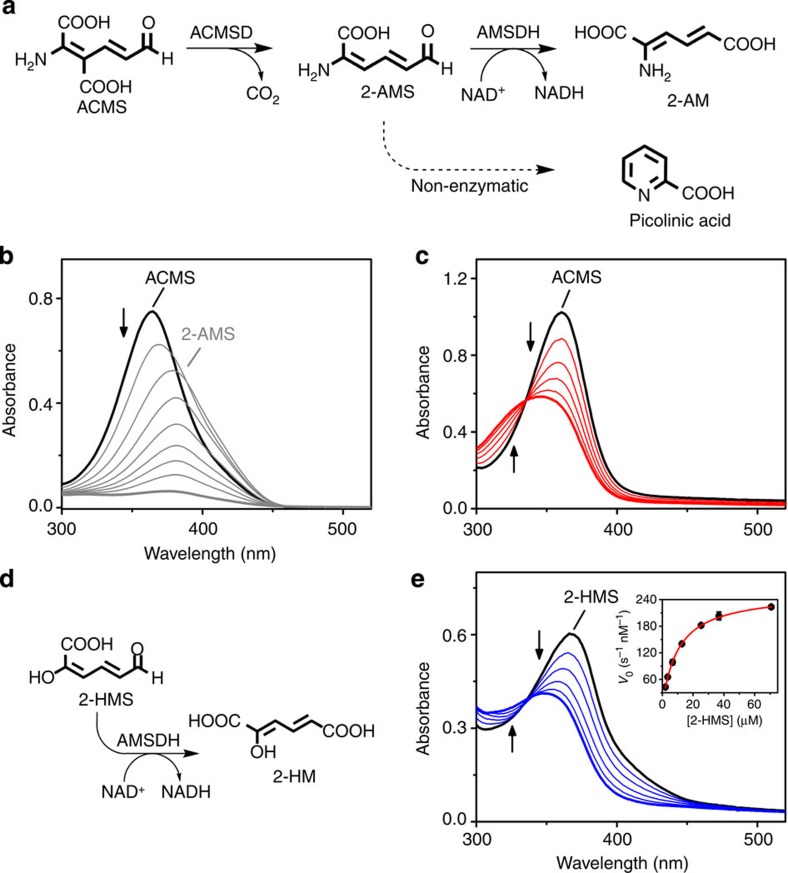
Activity of AMSDH. (**a**) Reaction scheme showing the enzymatic generation of 2-AMS, the reaction catalysed by AMSDH, and the competing non-enzymatic decay of 2-AMS to picolinic acid. (**b**) Representative assay showing the ACMSD (1 μM)-catalysed conversion of ACMS (*λ*_max_ 360 nm) to 2-AMS (*λ*_max_ 380 nm), which decays to picolinic acid (transparent). (**c**) Coupled-enzyme assay in which AMSDH (200 nM) oxidizes 2-AMS, produced *in situ* as shown in **b** in 50 s, to 2-AM (*λ*_max_ 325 nm). (**d**) Reaction scheme showing 2-HMS oxidation by AMSDH. (**e**) Representative assay showing the activity of AMSDH (200 nM) on 2-HMS (*λ*_max_ 375 nm) in 50 s. The inset is a Michaelis–Menten plot.

Due to the unstable nature of its substrate, 2-AMS, the activity of AMSDH was detected using a coupled-enzyme assay that employed its upstream partner, α-amino β-carboxymuconate ε-semialdehyde decarboxylase (ACMSD), to generate 2-AMS *in situ*. ACMSD transforms α-amino β-carboxymuconate ε-semialdehyde (ACMS) (*λ*_max_ at 360 nm) to 2-AMS (*λ*_max_ at 380 nm)^16^,^17^. As seen in Fig. 1b, in an assay that uses only ACMSD, the absorbance peak of its substrate, ACMS, red-shifts to 380 nm as 2-AMS is formed. The absorbance at 380 nm then quickly decreases as 2-AMS decays to picolinic acid, a compound with no absorbance features above 200 nm. In a coupled-enzyme assay, ACMSD, AMSDH and NAD^+^ are included in the reaction system. As shown in Fig. 1c, ACMS is still consumed; however, there is no red shift observed because 2-AMS is enzymatically converted to 2-AM (*λ*_max_ at 325 nm) rather than accumulating and decaying to picolinic acid. The production of 2-AM requires that an equimolar amount of NAD^+^ be reduced to NADH (*λ*_max_ at 339 nm). A stable alternative substrate, 2-hydroxymuconate-6-semialdehyde (2-HMS), was used to pursue kinetic parameters (Fig. 1d), when using saturating NAD^+^ concentrations (≥1 mM), the *k*_cat_ and *K*_m_ of AMSDH for 2-HMS were 1.30±0.01 s^−1^ and 10.4±0.2 μM, respectively (Fig. 1e).

### Structural snapshots of the dehydrogenase catalytic cycle

We solved five crystal structures of wild-type AMSDH, including the ligand-free (2.20 Å resolution), NAD^+^-bound binary complex (2.00 Å), ternary complex with NAD^+^ and substrate 2-AMS (2.00 Å) or 2-HMS (2.20 Å) and a thioacyl intermediate (1.95 Å). All five structures belong to space group *P*2_1_2_1_2_1_. Data collection and refinement statistics are listed in Supplementary Table 1. The complete AMSDH model includes four polypeptides per asymmetric unit describing one homotetramer (Supplementary Fig. 1a). Each monomer of AMSDH contains three domains: a subunit interaction domain, a catalytic domain and an NAD^+^ binding domain (Supplementary Fig. 1b). For details of the secondary structure, see Supplementary Discussion.

**Figure 2:**
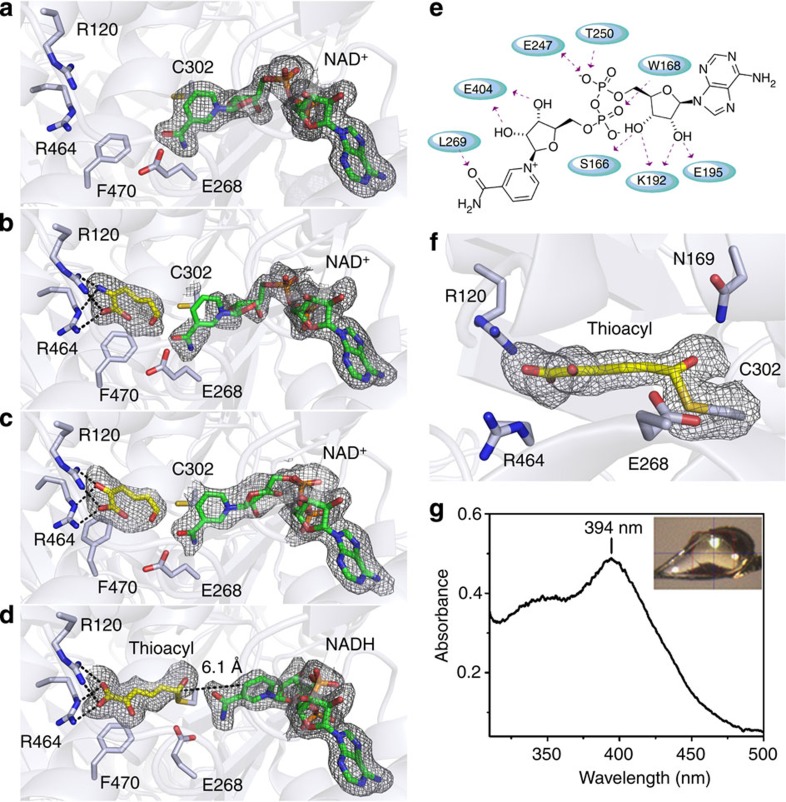
Crystal structures of wild-type AMSDH and single-crystal electronic absorption spectrum of a catalytic intermediate. AMSDH was co-crystallized with NAD^+^ to give AMSDH-NAD^+^ binary complex crystals that were used for soaking experiments. (**a**) Active site structure of the binary AMSDH-NAD^+^ complex, (**b**) the ternary complex of AMSDH-NAD^+^ crystals soaked with 2-AMS for 5 min before flash cooling, (**c**) the ternary complex of AMSDH-NAD^+^ soaked with 2-HMS for 10 min before flash cooling, (**d**) the trapped thioacyl, NADH-bound intermediate obtained by soaking AMSDH-NAD^+^ crystals with 2-HMS for 40 min before flash cooling. (**e**) Two-dimensional interaction diagram for NAD^+^ binding. (**f**) Close-up of the thioacyl intermediate in **d**. (**g**) Single-crystal electronic absorption spectrum of **d**. Protein backbone and residues are shown as light blue cartoons and sticks, respectively. The substrates and intermediate are shown as yellow sticks, and NAD^+^ and NADH are shown as green sticks. The omit map for ligands is contoured to 2.0 *σ* and shown as a grey mesh.

In the structure of the co-crystallized binary complex, an NAD^+^ molecule is present in an extended, anti-conformation in the amino-terminal, co-substrate-binding domain of each monomer (Fig. 2a). The electron density map of NAD^+^ is well defined, and the interactions between the protein and NAD^+^ are equivalent in all four subunits as shown in Fig. 2e. The NAD^+^-bound AMSDH structure is similar to the ligand-free structure with an aligned r.m.s.d. of 0.239 Å. Residues that belong to the NAD^+^-binding pocket are also well aligned with the exception of Cys302, Arg108 and Leu116 (Supplementary Fig. 2). On binding NAD^+^, the thiol moiety of Cys302 rotates so that the sulfur is 2.3 Å closer to the substrate-binding pocket and away from the nicotinamide head of NAD^+^.

### Crystal structures of enzyme–substrate ternary complexes

Structures of AMSDH in ternary complex with co-substrate NAD^+^ and its primary substrates were obtained by soaking co-crystallized AMSDH-NAD^+^ crystals with 2-AMS and 2-HMS, respectively. Extra density that fits with the corresponding substrate molecule was observed in the active site of each subunit. The co-substrate NAD^+^ in the ternary complex structures is bound in the same manner as in the binary complex. Binding of the primary substrates introduced minimal change to the protein structure; the r.m.s.d. for the superimposed structures of substrate-free with 2-AMS- and 2-HMS-bound ternary complex structures are 0.170 and 0.276 Å, respectively. These two primary substrates bind to AMSDH in an identical fashion, with two arginine residues, Arg120 and Arg464, playing an important role in stabilizing the substrate by forming two sets of bifurcated hydrogen bonds with one of the carboxyl oxygens and the 2-amino or hydroxyl group of 2-AMS (Fig. 2b) or 2-HMS (Fig. 2c), respectively. The observation of two hydrogen bonds being donated by the active site arginines to the 2-amino group of 2-AMS indicates that in the substrate-bound form, 2-AMS may be in its 2-imine rather than 2-enamine tautomer, as an amino group unlikely to accept two hydrogen bonds. Mutation of Arg120 to alanine causes a moderate decrease of the *k*_cat_ to 0.7±0.2 s^−1^ from 1.30±0.01 s^−1^ and a dramatic increase of the *K*_m_ with a lower bound of 446.3±195.9 μM (an accurate determination of the *K*_m_ is hindered by insufficient 2-HMS concentrations) compared with 10.4±0.2 μM in the wild type (Supplementary Fig. 3a). Mutation of Arg464 to alanine decreased the *k*_cat_ to ~0.3 s^−1^, and not only increased the *K*_m_ to ~170 μM, but also leads to a significant substrate inhibition effect with a *K*_i_ of ~6 μM (Supplementary Fig. 3b). This substrate inhibition is likely caused by the unproductive binding of a second substrate molecule in the space created by the deletion of Arg464 or by a failure of the enzyme to properly bind and stabilize the imine form of the substrate.

### Catalytic intermediates trapped after ternary complex formation

Enzyme–NAD^+^ binary complex crystals were soaked in mother liquor containing 2-HMS for a range of time points from 5 min to more than 3 h before flash cooling in liquid nitrogen. In a crystal that was soaked for 40 min, an intermediate was trapped and refined to a resolution of 1.95 Å (Fig. 2d). Crystals soaked for longer time points gave a similar intermediate with poorer resolution. In this structure, 2-HMS is observed in the 2*Z*, 4*E* isomer rather than the 2*E*, 4*E* isomer as seen in the substrate-bound ternary structure. Also, the substrate interacts with Arg120 and Arg464 with both of its carboxyl oxygens rather than one carboxyl oxygen and the 2-hydroxy oxygen as shown in the 2-HMS ternary complex structure. Fitting this density with the 2*E*, 4*E* conformation resulted in unsatisfactory 2*F*_o_−*F*_c_ and *F*_o_−*F*_c_ density maps as shown in Supplementary Fig. 4a. Likewise, attempting to fit the 2*Z*, 4*E* isomer to the ternary complex structure did not produce satisfactory results (Supplementary Fig. 4b). On *E* to *Z* isomerization, the carbon chain of the substrate extends, and the distance between its sixth carbon and Cys302’s sulfur is now at 1.8 Å, which is within covalent bond distance for a carbon–sulfur bond. Also, the continuous electron density between Cys302-SG and 2-HMS-C6 indicates the presence of a covalent bond (Fig. 2f). Another feature of this intermediate is that the nicotinamide ring of NAD^+^ has moved 4.6 Å away from the active site and adopted a bent conformation (Fig. 2d) compared with the position in the binary or ternary complex structures (Fig. 2a–c). The structural changes of NAD^+^ associated with reduction has been observed and well documented^18^,^19^. In the oxidized state, NAD(P)^+^ lies in the Rossmann fold in an extended conformation, allowing for hydride transfer from the substrate to its nicotinamide carbon during the first half of the reaction. Reduced NAD(P)H then adopts a bent conformation in which the nicotinamide head moves back towards the protein surface. This movement provides more space in the active site for the second half of the reaction, acyl-enzyme adduct hydrolysis, to take place. Thus, the coenzyme in this intermediate structure is likely to have been reduced to NADH and, as such, the structure is assigned as a thioacyl-enzyme–substrate adduct. The single-crystal electronic absorption spectrum of the sample has an absorbance maximum at 394 nm (Fig. 2g). The same absorbance band was observed in crystals soaked with 2-HMS from 30 min to 2 h (Supplementary Fig. 5a). However, this long-lived intermediate in the crystal was not observed in solution with millisecond-to-second time resolution in stopped-flow experiments (Supplementary Fig. 6a). Thus, it is either present in an earlier time domain (sub-milliseconds), or alternatively, it may not accumulate in solution because NADH can readily dissociate in solution, whereas it may be trapped in the active site when in the crystalline state.

**Figure 3:**
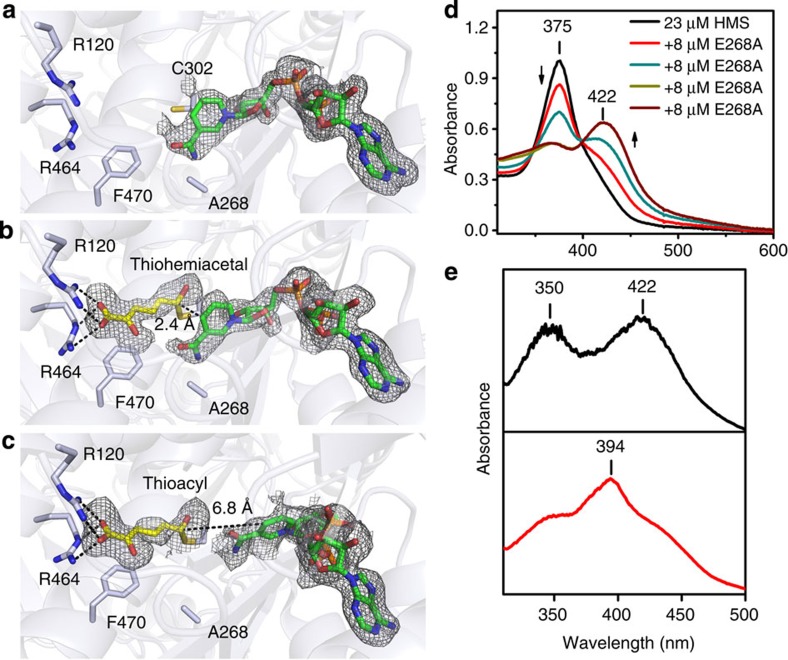
Crystal structures of the E268A mutant and its solution and single-crystal electronic absorption spectra. (**a**) Structure of the active site of the co-crystallized E268A-NAD^+^ binary complex, (**b**) a thiohemiacetal intermediate obtained by soaking the E268A-NAD^+^ crystals with 2-HMS for 30 min before flash cooling and (**c**) a thioacyl intermediate obtained by soaking the E268A-NAD^+^ crystals with 2-HMS for 180 min before flash cooling. (**d**) Solution electronic absorption spectra of a titration of 2-HMS with E268A. (**e**) Single-crystal electronic absorption spectrum of the intermediate in **b** (top panel) and single-crystal electronic absorption spectrum of the intermediate in **c** (bottom panel). Protein backbone and residues are shown as light blue cartoons and sticks, respectively. The substrate and intermediate are shown as yellow sticks, and NAD^+^ and NADH are shown as green sticks. The omit map for ligands is contoured to 2.0 *σ* and shown as a grey mesh.

**Figure 4:**
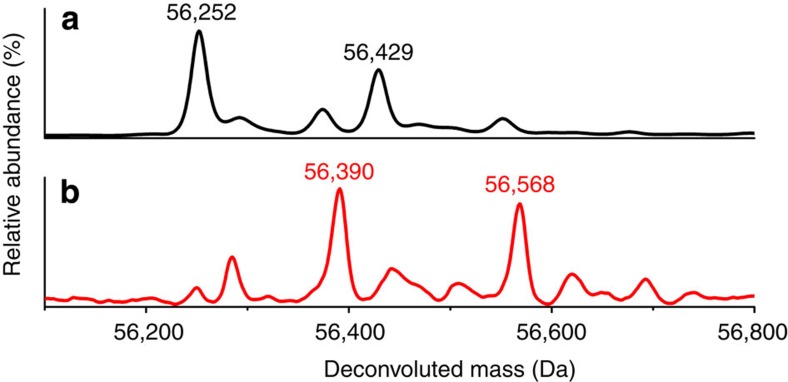
Deconvoluted positive-mode electrospray ionization mass spectra of as-isolated E268A (**a**) and 2-HMS treated-E268A (**b**). The two major components are labelled with their respective molecular weights.

Another notable change in the intermediate structure is the movement of the side chain of Glu268, which rotates 73° towards the active site (Fig. 2c,d). To probe the function of Glu268, we constructed an alanine mutant and found that it exhibited no detectable activity in steady-state kinetic assays. Interestingly, E268A exhibits completely different pre-steady state activity than the wild-type enzyme. As shown in Supplementary Fig. 6b, an absorbance band at 422 nm was formed concomitant with the decay of the 2-HMS peak within 1 s of the reaction. This new species is generated stoichiometrically on titration of 2-HMS with E268A (Fig. 3d). The moiety that gives rise to this new absorbance band is stable for minutes at room temperature and cannot be separated from the protein by membrane filtration-based methods^20^, suggesting that it is covalently bound to the protein. The formation of an enzyme–substrate adduct in the E268A mutant was investigated by mass spectrometry. For the as-isolated E268A, the resultant multiply charged states (Supplementary Fig. 7) were deconvoluted to obtain a molecular weight (MW) of 56,252 Da (Fig. 4a). This value is in good agreement with the predicted MW of E268A AMSDH plus an amino-terminal His-tag and linking residues, 56,251 Da. The second largest peak in the deconvoluted spectrum has a MW 177 Da greater than that of the most abundant signal. This is likely due to post-translational modification of the His-tag; α-*N*-Gluconoylation of His-tags has been observed in *E. coli*-expressed proteins, which cause 178 Da extra mass^21^. The mutant protein was then treated with the alternate substrate, 2-HMS, and the mass spectrum shows a new major peak at 56,390 Da (Fig. 4b), 138 Da heavier than the as-isolated mutant. Similarly, the second most abundant peak corresponds to a His-tag modified mutant plus 139 Da. In this spectrum, the peaks arising from the as-isolated mutant are substantially reduced, indicating that 2-HMS, 141 Da, is bound to the E268A mutant enzyme.

We determined the crystal structure of E268A co-crystallized with NAD^+^ and refined it to 2.00 Å resolution (Fig. 3a). The overall structure aligns very well with the wild-type binary complex structure with an r.m.s.d. of 0.139 Å. The active site of E268A also resembles the native AMSDH structure (Supplementary Fig. 8). The nature of the absorbing species at 422 nm was further investigated by soaking co-crystallized E268A-NAD^+^ crystals in mother liquor containing 2-HMS. By doing so, two temporally, structurally and spectroscopically distinct intermediates were identified.

**Figure 5:**
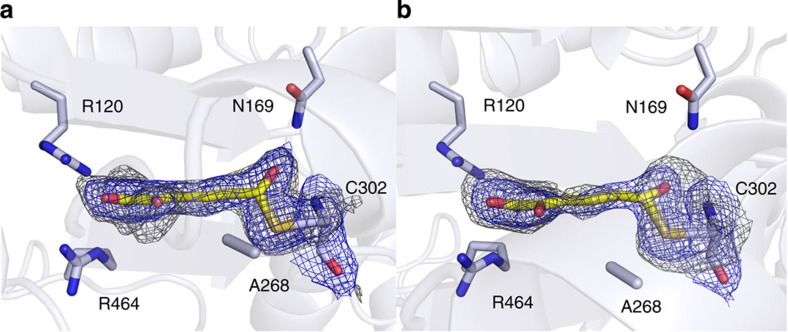
Crystal structures of two distinct catalytic intermediates. (**a**) Electron density map of the thiohemiacetal intermediate obtained from E268A-NAD^+^ crystal soaked with 2-HMS for 30 min. (**b**) Electron density map of the thioacyl intermediate obtained from E268A-NAD^+^ crystal soaked with 2-HMS for 180 min. The 2*F*_o_−*F*_c_ electron density map for ligands and Cys302 is contoured to 1.0 *σ* and shown as a blue mesh. The omit map for ligands and Cys302 is contoured to 2.0 *σ* and shown as a gray mesh.

When E268A-NAD^+^ crystals are soaked with 2-HMS for 40 min or less, their single-crystal electronic absorption spectra show an absorbance maximum at 422 nm (Supplementary Fig. 5b), as was observed in the solution-state titration and the stopped-flow assays. An individual electronic absorption spectrum for an E268A-NAD^+^ crystal soaked with 2-HMS for 15 min can be found in Fig. 3e (top). The structure of E268A-NAD^+^ soaked with 2-HMS for 30 min before flash cooling was solved and refined to 2.15 Å resolution (Fig. 3b). In this structure, a continuous electron density between Cys302-SG and 2-HMS-C6 is observed, similar to the thioacyl intermediate observed in the wild-type enzyme. However, in contrast to the thioacyl intermediate, the density around C6 is less flat, indicating an *sp*^3^- rather than *sp*^2^-hybridized carbon (Fig. 5a). The angle between the plane of the carbon backbone of the substrate and the formerly aldehydic oxygen is 55±9°, compared with the angle of the wild-type thioacyl intermediate at 26±4° (Supplementary Table 2). More importantly, the C6 of 2-HMS and the C4N of NAD^+^ are very close (2.4–2.8 Å), making it unlikely that the hydride has been transferred from the substrate. Taken together, these data allow us to assign this intermediate to a thiohemiacetal enzyme adduct (Fig. 3b). A similar intermediate has only been trapped once previously in a crystal that contains no co-substrate^22^. Hence, this is the first time for this intermediate to be trapped in the presence of NAD^+^.

If the E268A-NAD^+^ crystals are soaked with 2-HMS for longer than 1 h, their single-crystal electronic absorption spectra begin to resemble that of the wild-type, thioacyl intermediate with a corresponding absorbance maximum at 394 nm (Supplementary Fig. 5b), as seen in wild-type, thioacyl intermediate crystals. An individual electronic absorption spectrum for an E268A-NAD^+^ crystal soaked with 2-HMS for 120 min can be found in Fig. 3e (bottom). The structure of an E268A-NAD^+^ crystal soaked with 2-HMS for 180 min was solved and refined to 2.20 Å (Fig. 3c). The structure of this intermediate is also similar to the wild-type, thioacyl-enzyme adduct with NADH, rather than NAD^+^ found at the active site. The distance between the C4N of NADH and C6 of 2-HMS is longer than 6.1 Å (Fig. 3c). The electron density around C6 is flatter (Fig. 5b) compared with the thiohemiacetal intermediate and similar to the thioacyl intermediate trapped in the wild-type AMSDH structure (Fig. 2f), and the angle between the plane of the carbon backbone of the substrate and the carbonyl oxygen is 20±5°, which is statistically indistinguishable from that of the wild-type, thioacyl intermediate, 26±4° (Supplementary Table 2). On the basis of the similarities in their absorbance and structures, we conclude that this latter intermediate is equivalent to the wild-type, thioacyl intermediate. It is also worth noting that the strictly conserved asparagine 169 (Fig. 5) is seen to stabilize both the thiohemiacetal and thioacyl intermediates through hydrogen-bonding interactions.

### Investigation of isomerization by computational modelling

The isomerization of 2-AMS from the 2*E* to 2*Z* isomer implied by the solved crystal structures was probed with density functional theory calculations. The free energy profiles obtained were used to help illuminate the nature of 2-AMS and gain insight into how the active site of AMSDH may facilitate the isomerization. The total energies of different isomers and rotamers of 2-AMS in its enamine/aldehyde and imine/eneol tautomers and the rotational barriers about their respective 2–3 bond were compared. For the imine/eneol tautomer, additional computations were performed with the side groups from Arg120 and Arg464 to investigate what effect, if any, they will have on the free energy profile for rotation about the 2–3 bond of 2-AMS.

**Figure 6:**
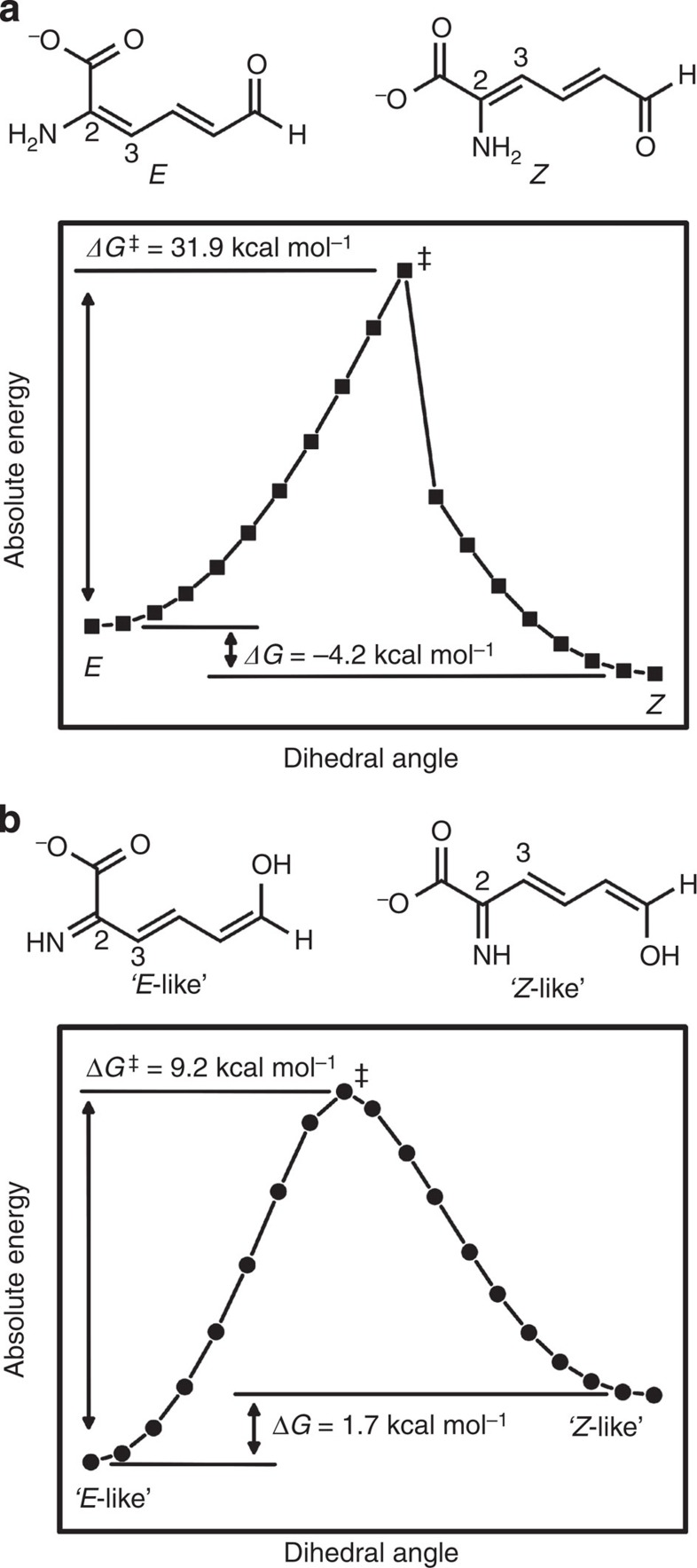
Free energy profiles for the rotation about the 2–3 bond of 2-AMS in its (**a**) enamine and (**b**) imine form, respectively. DFT calculations were performed at the B3LYP/6-31G*+ level of theory. The dihedral angle about the 2–3 bond was restrained in 10° increments and the structures were optimized at each point.

First, 2-AMS was constructed and optimized in its 2-enamine, 6-aldehyde, 2*E* isomer with a negatively charged 2-carboxylate group (Fig. 6a). To estimate the energy barrier for an uncatalysed isomerization from the 2*E* to the 2*Z* isomer, the 2–3 double bond was then restrained at 10° intervals from 180 to 0°, and the structure was optimized at each point. On the basis of the free energy profile (Fig. 6a), the uncatalysed isomerization barrier is 31.9 kcal mol^−1^. The profile also shows that the 2*Z* isomer, as might be expected, is lower in energy than the 2*E* isomer by 4.2 kcal mol^−1^. Next, the rotational barrier about the 2–3 bond of 2-AMS when in its 2-imine, 6-enol tautomer, as is suggested by the ternary complex structure, was calculated in the same manner. The barrier was found to be 9.2 kcal mol^−1^, and opposite to the enamine tautomer, the ‘2*Z*-like’ rotamer is higher in energy than the ‘2*E*-like’ rotamer by 1.7 kcal mol^−1^ (Fig. 6b). Unsurprisingly, the rotational barrier about the 2–3 bond is much lower in the imine tautomer; however, the ‘2*Z*-like’ rotamer of the imine tautomer is 21.8 kcal mol^−1^ higher in energy than the 2*Z* isomer of the enamine tautomer.

Possible influences of the two active site arginines on the free energy profile for rotation were also considered. To mimic the conditions of the enzyme active site, similar calculations as those above were performed, which included the guanidinium heads of Arg120 and Arg464. The starting model was built using the active site geometry of the ternary complex crystal structure (PDB entry: 4I25), and on inspection, it is immediately apparent that with two arginines in such close proximity to the substrate, there is insufficient space for two hydrogen atoms on the nitrogen at the 2-position of 2-AMS, and attempts to optimize an enamine tautomer with the hydrogen-bonding pattern of the ternary complex produced structures within which the entire 2-AMS molecule rotates so that only the carboxylate group interacts with the guanidinium moieties. The absolute positions of the guanidinium groups were fixed and the structure of 2-AMS in the imine tautomer was optimized. The dihedral angle of the 2–3 bond of 2-AMS was then increased in 45° increments and the structure optimized while restraining the position of the guanidinium groups and the 2–3 bond to build a rough free energy profile to estimate the rotational barrier. In the presence of the active site arginines, the barrier about the 2–3 bond of 2-AMS is further reduced to 8.5 kcal mol^−1^ (Supplementary Table 3). Another interesting finding is that in the presence of the guanidinium groups, the ‘2*E*-like’ and ‘2*Z*-like’ rotamers of 2-AMS are nearly isoenergetic, with a free energy difference of 0.2 kcal mol^−1^ (Supplementary Table 3).

## Discussion

The substrate of AMSDH, 2-AMS, contains an unstable aldehyde in conjugation with an enamine and can decay to picolinic acid and water, presumably through an electrocyclization reaction similar to its metabolic precursor, ACMS^23^. To assay the enzymatic activity, the upstream enzyme was utilized in the reaction mixture to generate substrate, and it was shown that AMSDH is catalytically active. Unfortunately, no kinetic parameters can be reliably determined because the concentration of 2-AMS is not well defined in the coupled-enzyme assay. To circumvent this issue, a previously-identified, stable alternative substrate, 2-HMS^24^,^25^, in which a hydroxyl group replaces the amino group in 2-AMS to prevent cyclization, was used to characterize the activity of AMSDH and to examine the activity of the mutants.

Substrate-bound, ternary complex structures were obtained by soaking co-crystallized protein and NAD^+^ with 2-AMS or 2-HMS. 2-AMS is an unstable compound which decays with a *t*_1/2_ of about 9 s at pH 7.5 and 37 °C or 35 s at pH 7.0 and 20 °C. Notably, this is its first reported structure. It appears to be stabilized in the enzyme active site in its imine tautomer by forming two sets of bifurcated hydrogen bonds with Arg120 and Arg464 so that the electrocyclization reaction cannot occur. Both arginine residues are close to the protein surface and in good positions to serve as gatekeepers, bringing the substrate into the active site. As a residue residing on a loop, Arg464 should be relatively flexible. The electron density for the side chain of Arg120 is partially missing in the binary complex structure but very well resolved in both ternary complex structures. This observation indicates that the presence of substrate can stabilize what may be a flexible residue. It becomes evident from the coordinates that Arg120 and Arg464 play an important role in substrate recognition, stabilization and possibly product release. Two arginine residues are rarely observed in such close proximity, stabilizing one end of the same molecule with multiple hydrogen bonds. With the exception of the hydrogen bonds provided by Arg120 and 464, the substrate-binding pocket is mostly composed of hydrophobic residues. On the basis of sequence alignment (Supplementary Fig. 9), these two arginine residues are strictly conserved throughout the HMSDH family but are not found in other members of the ALDH superfamily. We propose that these dual arginines combined with the size restrictions provided by the hydrophobic pocket endow this enzyme with its specificity towards small α-substituted carboxylic acids with an aldehyde moiety, such as 2-AMS and 2-HMS. Furthermore, our computational work suggests that these arginines are crucial for stabilizing the imine tautomer of 2-AMS to allow for rotation about its 2–3 bond.

Two strictly conserved catalytic residues, Cys302 and Glu268, are present at the interior of the substrate-binding pocket. General features regarding these residues in the ALDH superfamily are (1) that the cysteine serves as a catalytic nucleophile, which is anticipated to form a covalent-adduct intermediate with the substrate by a nucleophilic addition during catalysis^26^,^27^,^28^ and (2) that the glutamate serves as a base to activate water for hydrolysis of the thioacyl-enzyme adduct^29^,^30^,^31^,^32^. Previous studies indicate that the catalytic cysteine can adopt two conformations, resting and attacking^19^. In the ligand-free structure, Cys302 is far from where the carbonyl carbon of the substrate should be and is in the resting state. In the ternary complex structures, Cys302 is located at an ideal position to initiate catalysis, which is the attacking state. It is proposed to attack the aldehydic carbon (C6) of the substrate. In the two ternary complex structures, the distance between the sulfur of Cys302 and the C6 of the substrate is ~3.3 Å. Cys302 and the aldehydic carbon form a covalent bond in both thioacyl and thiohemiacetal intermediates. Mutation of Cys302 to serine led to enzyme with no detectable dehydrogenase activity, further confirming its catalytic significance.

Examining the wild-type AMSDH structures shows that in the NAD^+^-bound binary complex, Glu268 adopts a ‘passive’ conformation, pointing away from the substrate-binding pocket, and forms hydrogen bonds with both NE of Trp177 (3.2 Å distance) and the backbone oxygen of Phe470 (3.6 Å) to leave space for the reduction of NAD^+^. Its electron density is very well resolved and the side chain B-factor is close to average: 28.2 Å^2^/28.5 Å^2^. The thiol moiety of Cys302 is 7.14 Å from Glu268 and is unlikely to form interactions. Interestingly, in both substrate-bound structures, Glu268 becomes more flexible and exhibits much weaker electron density and increased side chain B-factors compared with average protein B-factors: 37.8 Å^2^/28.5 Å^2^ and 66.37 Å^2^/39.7 Å^2^. In the thioacyl intermediate structure, the electron density of Glu268 becomes very well defined again, but its side chain rotates 73° towards the bound substrate and seems to be in an ‘active’ position to abstract a proton from a deacylating water (Fig. 2d). At this point in the reaction cycle, the NADH molecule needs to leave the active site to make room for the catalytic water molecule. Movement of the nicotinamide ring of NAD^+^ coupled with the rotation of an active site glutamate has previously been observed in other ALDHs during catalysis^30^,^31^,^32^.

Mutation of Glu268 to alanine led to the accumulation of the thiohemiacetal intermediate in both solution and crystalline states. The strictly conserved glutamate residue in the active site of ALDH enzymes has been proposed to play up to three possible roles during catalysis. It is strictly required to activate the deacylating water that allows for product release, it is in a ‘passive’ conformation during NAD(P)^+^ reduction, and in some cases, it may serve to activate cysteine for nucleophilic attack^33^. On the basis of these roles, mutation to alanine would be expected to decrease the rate of hydrolysis of the thioacyl adduct, have no effect on the rate of reduction of NADH and possibly decrease the rate of nucleophilic attack by cysteine. With this understanding, deletion of the active site glutamate should cause an accumulation of the thioacyl intermediate. However, in this work, the E268A mutant is shown to accumulate the preceding thiohemiacetal intermediate both in crystal and in solution. This finding suggests an additional catalytic role for this residue: rotation of Glu268 towards the active site facilitates the hydride transfer from the tetrahedral thiohemiacetal adduct to NAD^+^. The rapid formation of the intermediate in solution indicates that Glu268 of AMSDH does not play a role in activating cysteine. However, it does appear necessary to complete hydride transfer from the substrate to NAD^+^, and its removal turns the native, primary substrate into a suicide inhibitor.

**Figure 7:**
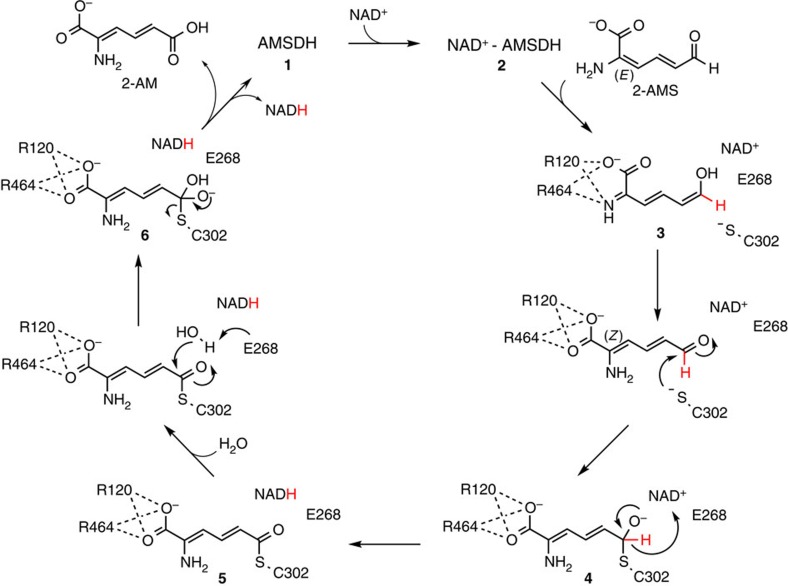
Proposed catalytic mechanism for the oxidation of 2-AMS by AMSDH. The primary substrate (2*E*, 4*E*)-2-aminomuconate-semialdehyde binds to the enzyme in its imine tautomer to form the ternary complex (**3**). An isomerization and attack by cysteine on the aldehydic carbon form the (2*Z*, 4*E*)-2-aminomuconate-thiohemiacetal adduct (**4**). AMSDH-mediated oxidation of **4** concomitant with reduction of NAD^+^ to NADH follows, generating a thioacyl-enzyme intermediate (**5**). Both **4** and **5** are the catalytic intermediates covalently attached to the enzyme. Hydrolysis of **5** then allows the release of the products 2-AM and NADH, restoring the ligand-free enzyme for the next catalytic cycle.

On the basis of previous studies of the ALDH mechanism, the eight high-resolution crystal structures solved (Supplementary Table 1) as well as our biochemical and computational studies, we propose a catalytic mechanism for AMSDH. As shown in Fig. 7, NAD^+^ binds to the enzyme, **1**, to form an NAD^+^-bound AMSDH complex, **2**. The substrate, 2-AMS, is then recognized by Arg120 and Arg464 through multiple hydrogen-bonding interactions, and its imine tautomer is stabilized in the active site, **3**. At this point, the order of the rotation, tautomerization and nucleophilic attack by C302 on the aldehydic carbon to produce the tetrahedral, thiohemiacetal intermediate, **4**, is not yet clear. The isomerization and nucleophilic attack drive a translation of the substrate away from Arg120 and Arg464 so that they are only able to interact with the carboxylate group of the substrate. Next, NAD^+^ is reduced to NADH by abstraction of a hydride from **4**, forming a thioacyl intermediate, **5**, a process which involves an *sp*^3^-to-*sp*^2^ transition during oxidation of the organic substrate by NAD^+^. On reduction, the nicotinamide portion of NADH moves away from the substrate as Glu268 rotates into position to activate a water molecule to perform a nucleophilic attack on the same carbon that was previously attacked by Cys302, forming a second tetrahedral intermediate, **6**. Finally, the second tetrahedral intermediate collapses, breaking the C–S bond and releasing the final products, 2-AM and NADH. Species **1**–**5** are spectroscopically and structurally characterized, while intermediate **6** was not seen to accumulate.

In this work, five catalytically relevant structures of the wild-type AMSDH and three mutant structures yield a comprehensive understanding of the protein’s overall structure, co-substrate-binding mode and elucidate the primary residues responsible for substrate specificity among the HMSDH family of the ALDH superfamily. The structural and spectroscopic snapshots capture the crystal structure of an unstable kynurenine metabolite, 2-AMS, and two catalytic intermediates, including stabilizing a tetrahedral intermediate in a mutant protein, which was further verified by mass spectrometry. Capture of a thiohemiacetal intermediate upon deletion of E268 also points to a new role for this well-established active site base in hydride transfer from the substrate to NAD^+^. Another interesting finding revealed through solving the ternary complex and intermediate crystal structures and supported by computational studies is that an *E* to *Z* isomerization of the substrate occurs in this dehydrogenase before hydride transfer. To the best of our knowledge, this is the first piece of structural evidence illustrating an ALDH that proceeds via an *E*/*Z* isomerization of its substrate during catalysis.

## Methods

### General methods

The cloning, expression, purification and site-directed mutagenesis of AMSDH are described in the Supplementary Methods.

### Preparation of ACMS and 2-HMS

ACMS was generated by catalysing the insertion of molecular oxygen to 3-hydroxyanthranilic acid by purified, Fe^2+^ reconstituted 3-hydroxyanthranilate 3,4-dioxygenase as described previously^16^,^20^. 2-HMS is generated non-enzymatically from ACMS following a previously established method^24^. The pH of solutions containing ACMS was adjusted to ~2 by the addition of hydrochloric acid. 2-HMS formation was monitored on an Agilent 8453 diode-array spectrophotometer at 315 nm. The solutions were then neutralized with sodium hydroxide once the absorbance at 315 nm stopped increasing. 2-HMS at neutral pH has a maximum absorbance at 375 nm (ref. 24).

### Enzyme activity assay using 2-HMS as substrate

Steady-state kinetics analyses were carried out at room temperature on an Agilent 8453 diode-array spectrophotometer. Reaction buffer contains 25 mM HEPES and 1 mM NAD^+^, pH 7.5. Consumption of 2-HMS by 200 nM AMSDH was detected by monitoring the decrease of its absorbance at 375 nm with a molar extinction coefficient of 43,000 M^−1^cm^−1^ (ref. 24) for 15 s with a 0.5 s integration time. For mutants, 700 nM protein and a wavelength of 420 nm, *ε*_420_ 11,180 M^−1^cm^−1^, was used. Absorbance at 375 nm decreased and blue shifted to 295 nm, the maximum ultraviolet absorbance for the product, 2-hydroxymuconic acid. This is consistent with previous reports in which the ending compound was purified and verified as the correct product^24^. The pre-steady state spectra were obtained with an Applied Photophysics Stopped-Flow Spectrometer SX20 (UK) with the mixing unit hosted inside an anaerobic chamber made by Coy Laboratory Products (MI, USA). Pre-steady state activity used the same reaction buffer but with 23 μM AMSDH or E268A and 25 μM 2-HMS and were carried out at 10 °C. The change in absorbance was monitored for 1.0 s.

### X-ray crystallographic data collection and refinement

Purified AMSDH samples at a final concentration of 10 mg ml^−1^ containing no NAD^+^ or 10 equiv. of NAD^+^ were used to set up sitting-drop vapour diffusion crystal screening trays in Art Robbins 96-well Intelli-Plates using an ARI Gryphon crystallization robot. The initial crystallization conditions were obtained from PEG-Ion 1/2 (Hampton Research) screening kits at room temperature. The screened conditions were optimized by increasing protein concentration to 40 mg ml^−1^ and lowering crystallization temperature to 18 °C. NAD^+^-bound AMSDH crystals were obtained from drops assembled with 1.5 μl of protein (preincubated for 10 min with 10 equiv. of NAD^+^) mixed with 1.5 μl of a reservoir solution containing 20% polyethylene glycol 3350 and 0.2 M sodium phosphate dibasic monohydrate, pH 9.1, by hanging drop diffusion in VDX plates (Hampton Research). Pyramid shaped crystals that diffract up to ~1.9 Å appeared overnight. The reservoir solution for crystallizing the cofactor-free AMSDH crystals contains 12% polyethylene glycol 3350, 0.1 M sodium formate, pH 7.0. Crystals belonging to the same space group formed within 2–3 days with an irregular plate shape and diffracted up to ~2.2 Å. NAD^+^-AMSDH crystals were used for substrate-soaking experiments. Crystals were transferred to mother liquor solution containing ~1 mM 2-HMS and incubated for 10–180 min before flash cooling in liquid nitrogen. Soaking 2-AMS as a substrate is more complicated because of its instability. Crystallization solution containing ~1.5 mM ACMS were used for soaking. After transferring several crystals to the soaking solution (8 μl), 2 μl of 1 mM purified ACMSD was included to catalyse the conversion of ACMS to 2-AMS. Crystals were flash frozen after a 5 min-incubation. Crystallization solution containing 20% glycerol or ethylene glycol was used as cryoprotectant. X-ray diffraction data were collected on SER-CAT beamline 22-ID or 22-BM of the Advanced Photon Source, Argonne National Laboratory.

### Ligand refinement and molecular modelling

The first AMSDH structure, the cofactor NAD^+^ bound structure, was solved by the molecular replacement method with the Advanced Molecular Replacement coupled with Auto Model Building programs from the PHENIX software using 5-carboxymethyl-2-hydroxymuconate semialdehyde dehydrogenase (PDB: 2D4E) as a search model, which shares 39% of amino-acid sequence identity with *P. fluorescens* AMSDH. The ligand-free, mutant and ternary complex structures were solved by molecular replacement using the refined NAD^+^-AMSDH as the search model. Refinement was conducted using PHENIX software^34^. The program Coot was used for electron density map analysis and model building^35^. NAD^+^/NADH, substrates 2-AMS and 2-HMS and Cys-substrate covalent-adduct intermediate were well defined and added to the model based on the 2*F*_o_−*F*_c_ and *F*_o_−*F*_c_ electron density maps. Refinement was assessed as complete when the *F*_o_−*F*_c_ electron density contained only noise. The structural figures were generated using PyMOL software (http://www.pymol.org/).

### Single-crystal spectroscopy

Electronic absorption spectra from single crystals held at 100 K were collected at beamline X26-C of the National Synchrotron Light Source (NSLS)^36^. The electronic absorption data were typically obtained between 200 and 1,000 nm with a Hamamatsu (Bridgewater, N.J.) L10290 high-power ultraviolet–visible light source. The lamp was connected to one of several 3-m long solarization-resistant optical fibres with an internal diameter of 115, 230, 400 or 600 μm (Ocean Optics, Dunedin, FL). The other end was connected to a 40-mm diameter, 35 mm working distance 4 × , Schwardchild design reflective microscope objective (Optique Peter, Lentilly France). The spectroscopy spot size is a convolution of the optical fibre diameter and the magnification of the objective, which in this case produced 28, 50, 100 or 150 μm diameter spots, respectively. Photons that passed through the crystal were collected with a second, aligned objective that was connected to a similar optical fibre or one with a slightly larger internal diameter. The spectrum was then recorded with either an Ocean Optics USB4000 or QE65000 spectrometer. Anisotropic spectra and an image of the crystal/loop were collected as a function of rotation angle in 5° increments. These were analysed by XREC^37^ to determine the flat face and optimum orientation.

### Mass spectrometry

To prepare samples for ESI mass spectrometry, as-isolated E268A AMSDH was buffer-exchanged to 10 mM Tris (pH 8.0) by running through a desalting column (GE Healthcare). Intermediate bound E268A was obtained by mixing E268A with 3 equiv. of 2-HMS. Excess 2-HMS was removed by desalting chromatography using the same buffer. Desalted proteins were concentrated to a final concentration of 20 μM. Freshly prepared samples were rinsed by acetonitrile and 0.1% formic acid (1:1 ratio) before injection. Mass spectrometry experiments were conducted using a Waters (Milford, MA) Micromass Q-TOF micro (ESI-Q-TOF) instrument operating in positive mode. The capillary voltage was set to 3,500 V, the sample cone voltage to 35 V and the extraction cone voltage to 2 V. The source block temperature and the desolvation temperature were set to 100 and 120 °C, respectively. The samples were introduced into the ion source by direct injection at a flow rate of 5 μl min^−1^. The raw data containing multiple positively charged protein peaks were deconvoluted and smoothed using MassLynx 4.1.

### Computational studies

All ground-state density functional theory calculations were performed with Gaussian 03 Revision-E.01 at the B3LYP/6-31G*+ level of theory^38^. The chemical structures were optimized using the ternary complex crystal structure (PDB entry: 4I25) as a starting model. To calculate the isomerization barrier, the dihedral angle about the 2–3 bond was restrained and the rest of the molecule was optimized. For the calculations that included the guanidinium heads of Arg120 and Arg464, the geometry was obtained from the crystal structure, and their positions were fixed while the substrate was optimized.

## Additional information

**How to cite this article:** Huo, L. *et al*. Crystallographic and spectroscopic snapshots reveal a dehydrogenase in action. *Nat. Commun.* 6:5935 doi: 10.1038/ncomms6935 (2015).

**Accession codes:** Coordinates and structure factors for apo-AMSDH, NAD^+^-bound AMSDH, NAD^+^- and 2-AMS-bound AMSDH, NAD^+^- and 2-HMS-bound AMSDH, thioacyl intermediate AMSDH, E268A AMSDH, E268A thiohemiacetal intermediate, and E268A thioacyl intermediate have been deposited in the RCSB Protein Data Bank under accession codes 4I26, 4I1W, 4I25, 4I2R, 4NPI, 4OE2, 4OU2, and 4OUB, respectively.

## Supplementary Material

Supplementary Information Supplementary Figures 1-9, Supplementary Tables 1-3, Supplementary Discussion, Supplementary Methods and Supplementary References
