# Supplementary material for: Crystallographic and spectroscopic snapshots reveal a dehydrogenase in action
Source: Nat Commun. 2015 Jan 7;6:5935. doi: 10.1038/ncomms6935 (PMC4286809; doi:10.1038/ncomms6935)
Supplement: Supplementary Information — Supplementary Figures 1-9, Supplementary Tables 1-3, Supplementary Discussion, Supplementary Methods and Supplementary References [file ncomms6935-s1.pdf]

Supplementary Figures

Supplementary Figure 1. Overall crystal structure of AMSDH. (**a**) View of the tetramer with the subunit A in pink, subunit B in blue, subunit C in yellow, and subunit D in green. Each subunit contains an NAD^+ molecule, which is shown in stick representation and colored by elements. (**b**) Structure of one AMSDH subunit with NAD^+ and substrate 2-AMS. The ribbon trace is rainbow-colored with the N-terminus in blue and the C-terminus in red. Top right is the subunit interaction domain, bottom right shows the cofactor binding domain, and top middle and left is the catalytic domain. NAD^+ and 2-AMS are shown as stick models with gray color. (**c**) Topology diagram showing the AMSDH secondary structure, which is also rainbow-colored according to cartoon in (**b**).

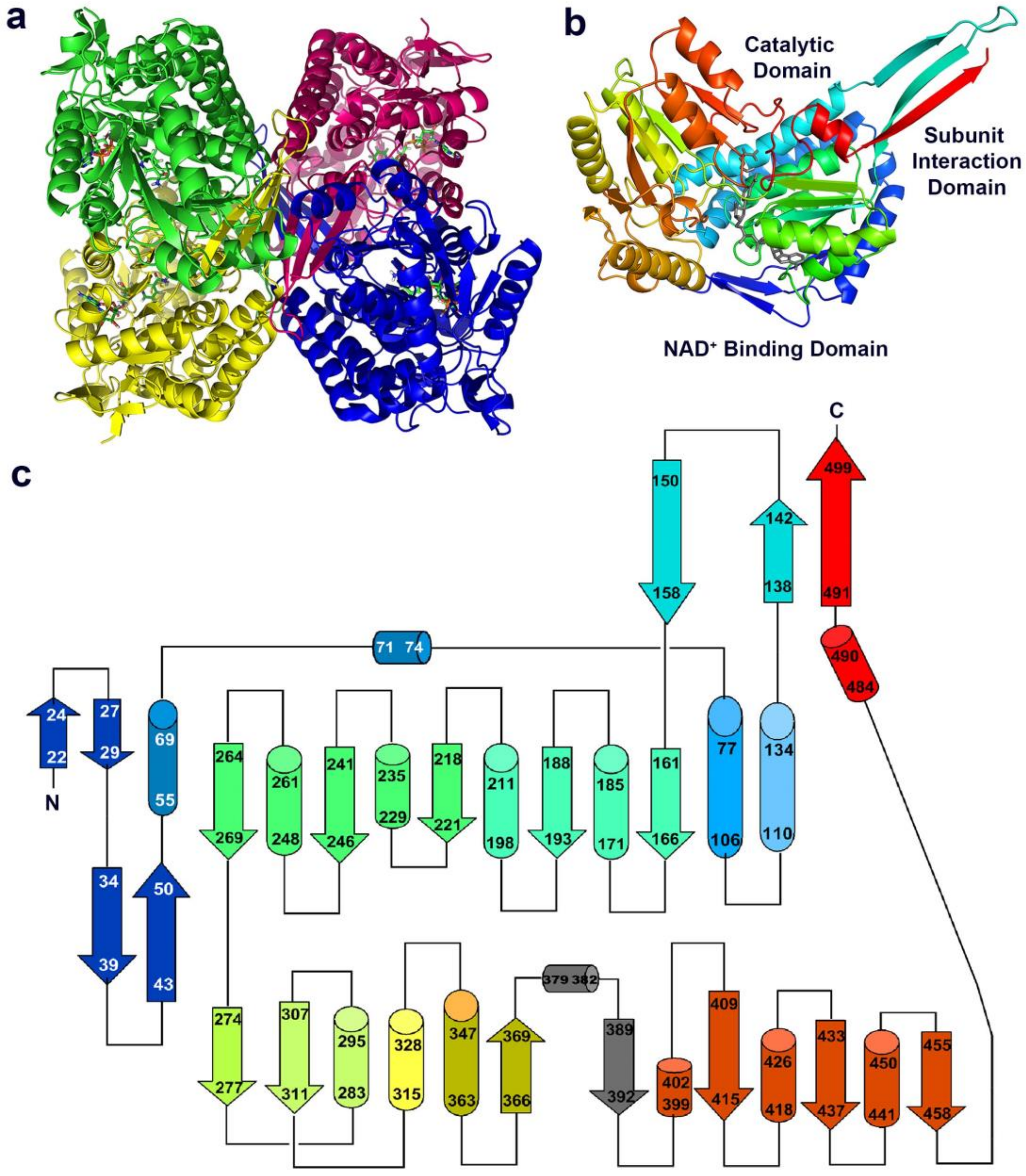

Supplementary Figure 2. Local changes at the active site of AMSDH induced by NAD$^+$ binding. Superimposition of the active sites of apo-AMSDH (yellow) and NAD$^+$-bound-AMSDH (blue). The $2F_o$ - F_c electron density map is contoured to 1.0 σ and shown as a grey mesh. Residues and NAD$^+$ are shown as sticks. The overall structure aligned very well with a RMSD of 0.239 Å. Arg108, Val116, and Cys302 are the only residues to have notable conformational changes caused by NAD$^+$ binding.

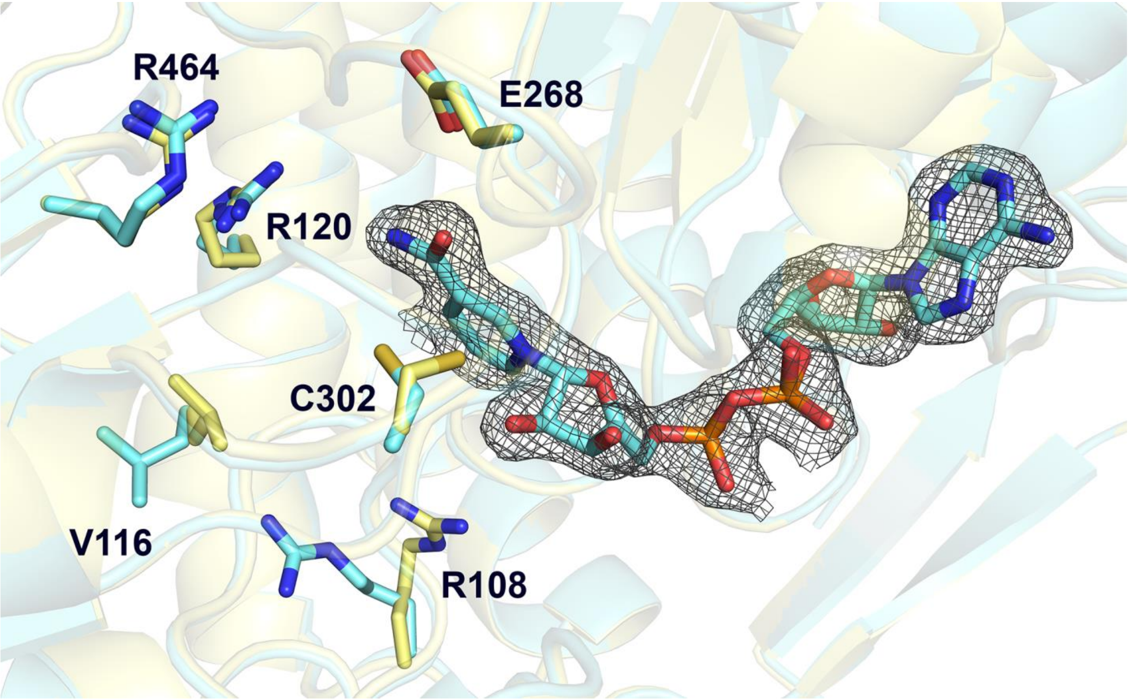

Supplementary Figure 3. Kinetic assays of R120A and R464A with 2-HMS. (a) Activity of R120A fit with the Michaelis-Menten equation: $v_0 = (V_{max} \times [S])/(K_m + [S])$; **(b)**, activity of R464A fit with the Michaelis-Menten equation with substrate inhibition:

$$v_0 = (V_{max} \times [S])/(K_m + [S] + [S]^2/K_i)$$

v_0 is the initial rate, $V_{\max}$ is the maximum rate, $[S]$ is the substrate concentration, K_{m} is the Michaelis constant, and K_{i} is the substrate inhibition constant. Assay details can be found in the materials and methods.

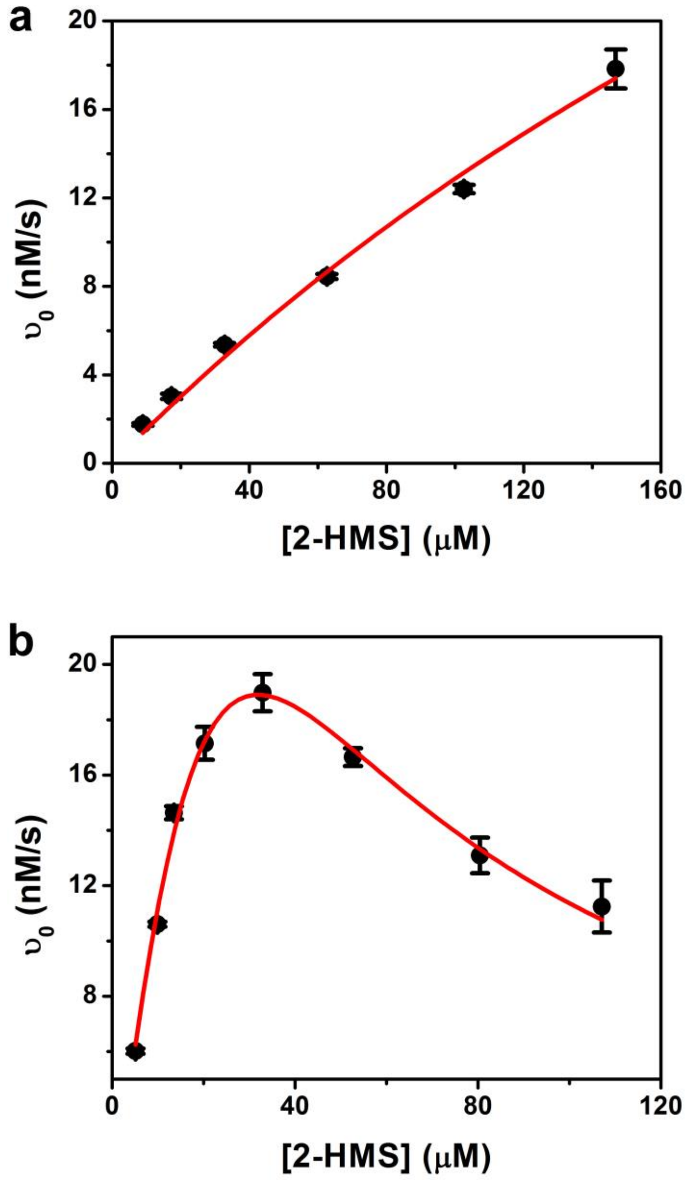

Supplementary Figure 4. Alternate fitting of substrate-bound ternary complex and thioacyl intermediate. (a) Electron density map of the thioacyl intermediate fit with incorrect model of (2*E*, 4*E*)-2-hydroxy-6-oxohex-2,4-enoic acid (left) and the correct model (right), respectively. **(b)** Electron density map of the substrate-bound ternary complex fit with incorrect model of (2*Z*, 4*E*)-2-hydroxy-6-oxohex-2,4-enoic acid (left) and the correct model (right), respectively. The $2F_o$ - F_c map is shown in gray mesh contoured at 1.0 σ. The F_o - F_c electron density maps are shown in green mesh contoured at 3.0 σ and red mesh contoured at -3.0 σ. The chemical structure of each ligand is shown on the left of the fitting.

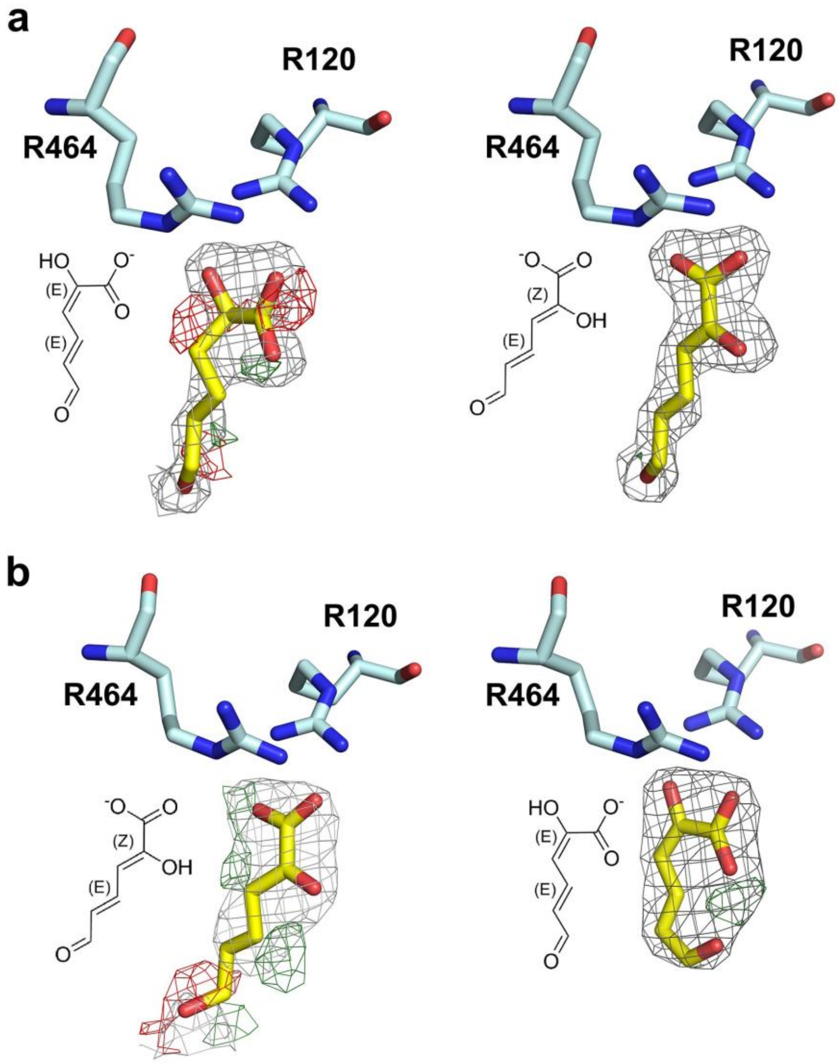

Supplementary Figure 5. Single-crystal electronic absorption spectra of wtAMSDH and E268A AMSDH co-crystallized with NAD^+ and soaked with 2-HMS before flash-cooling in liquid nitrogen. (**a**) wtAMSDH co-crystallized with NAD^+ and soaked with 2-HMS for increasing time. (**b**) Same experiment as **a** using E268A AMSDH. The soaking solution contains 0.2 M sodium phosphate dibasic, pH 9.1, 20% polyethylene glycol 3350, and 1 mM 2-HMS.

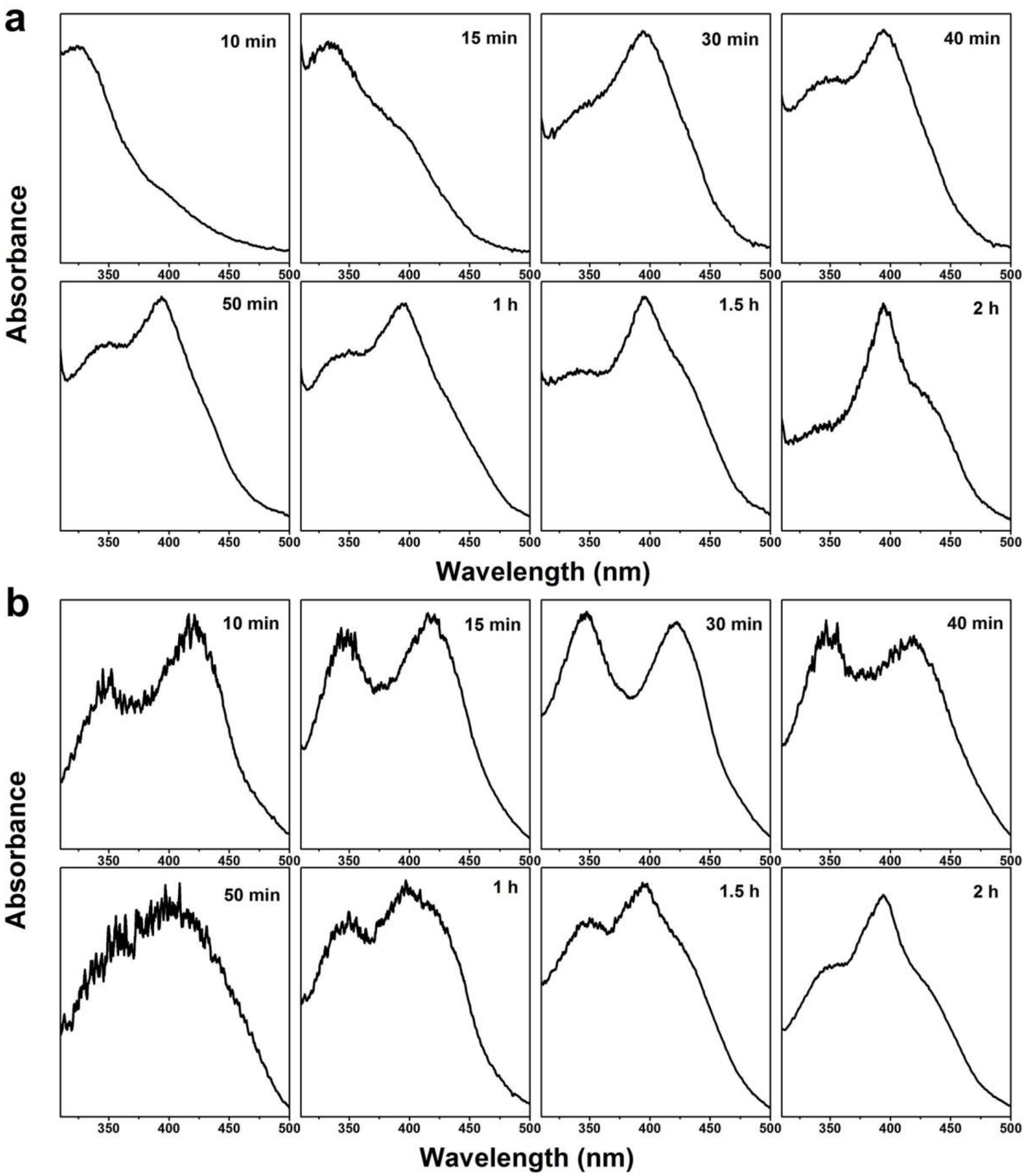

Supplementary Figure 6. Time-resolved, stopped-flow UV-Vis spectra of the reactions catalyzed by wtAMSDH and E268A. (**a**) wtAMSDH (23 μM) is mixed with 2-HMS (25 μM) and observed for 1.0 s. Decay of 2-HMS (λ_{max} 375 nm) is inset. (**b**) E268A AMSDH (23 μM) is mixed with 2-HMS (25 μM) and observed for 1.0 s. Decay of 2-HMS (λ_{max} 375 nm) and the formation of an intermediate (λ_{max} 422 nm) are inset. The reaction is performed in buffer containing 25 mM HEPES, pH 7.5, and 1 mM NAD^+ at 10 °C. The arrows indicate the trends of changes in the spectra.

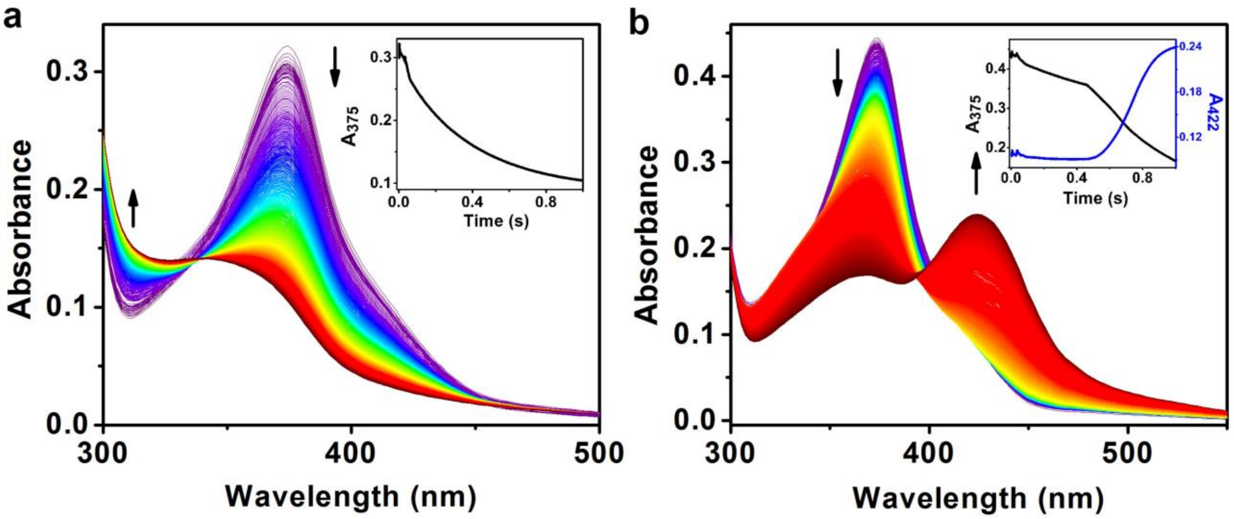

Supplementary Figure 7. Raw ESI mass spectra of as-isolated E268A AMSDH **(a)** and E268A treated with 2-HMS **(b)**. Narrow range scans corresponding to the proteins are shown in the insets.

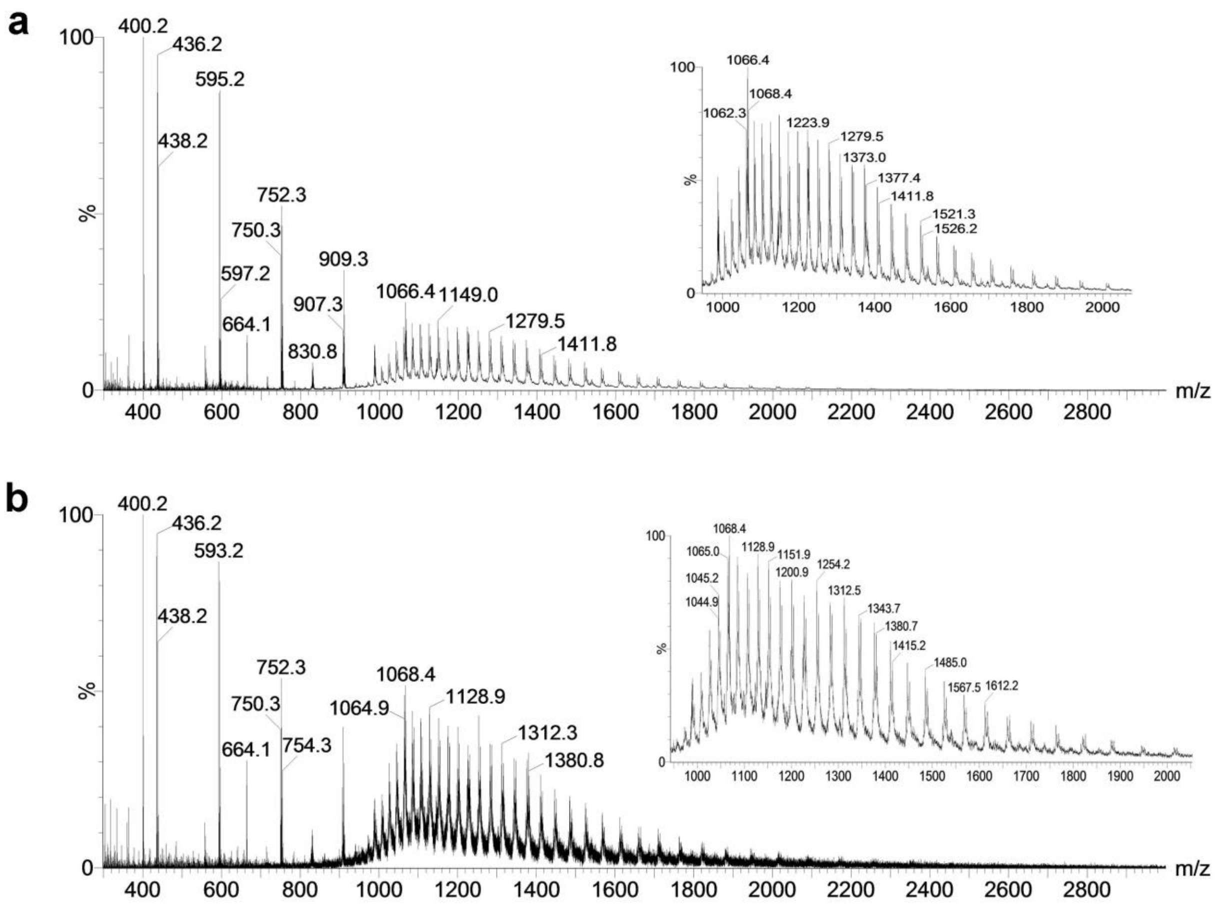

Supplementary Figure 8. Superimposition of E268A (pink) active site with wtAMSDH (light blue). Cys302, Glu/Ala 268 and NAD^+ are present as sticks. The $2F_o$-F_c electron density map of Ala268 is shown as gray mesh contoured at 1.0 σ.

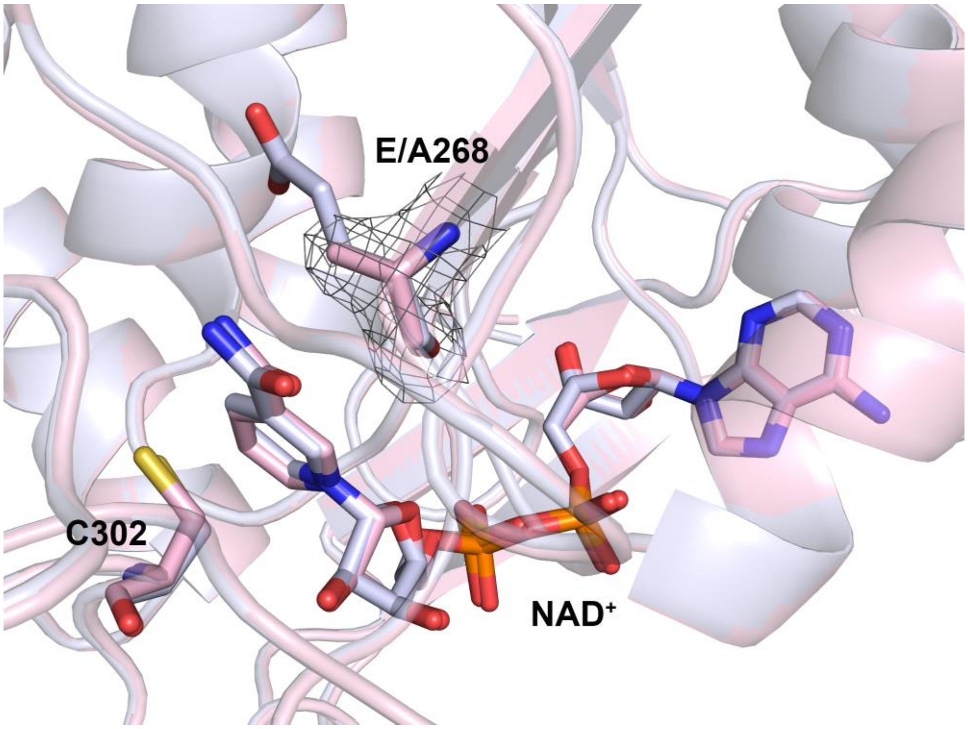

Supplementary Figure 9. Identification of substrate binding residues for the hydroxymuconic semialdehyde dehydrogenase (HMSDH) family. Sequence alignment of several enzymes from the HMSDH family of aldehyde dehydrogenase superfamily: AMSDH from *Psuedomonas fluorescens* (gene ID: 28971621), AMSDH from *Cupriavidus metallidurans* (gene ID: 94314125), HMSDH from *Cupriavidus basilensis* (gene ID: 493151182), HMSDH from *Pseudomonas sp.* M1 (gene ID: 575528385), ACSDH from *Comamonas testosterone* (gene ID: 190571970), CHSDH from *Glaciecola arctica* (gene ID: 494892710). Highly conserved residues are shown with red text and boxed in blue, strictly conserved residues are shown with a red background, conserved arginines for substrate binding are shown with a green background, the catalytic cysteine and glutamate are shown with a blue background, and hydrophobic residues in the active-site pocket are shown with pink background. This figure was prepared using ESPript [3].

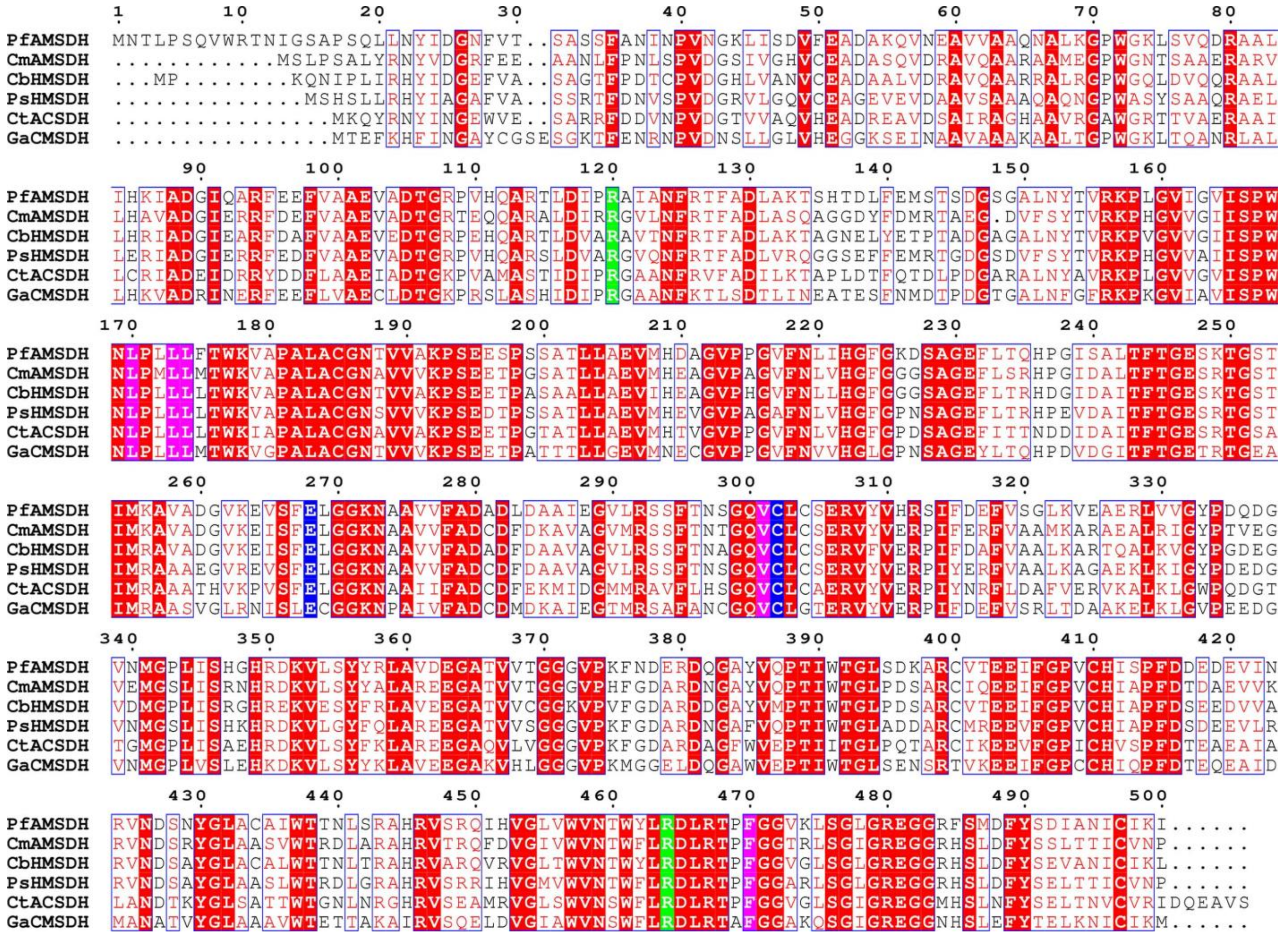

Supplementary Table 1. X-ray crystallography data collection and refinement statistics.

Data collection	Apo-AMSDH	NAD-AMSDH	NAD-2-AMS-AMSDH	NAD-2-HMS-AMSDH	Thioacyl Intermediate	E268A-AMSDH	E268A-Thiohemiacetal Intermediate	E268A-Thioacyl Intermediate
detector type	MAR300 CCD	MAR225 CCD	MAR300 CCD	MAR300 CCD	MAR225 CCD	MAR225 CCD	MAR225 CCD	MAR225 CCD
source	APS, Sector 22-ID	APS, Sector 22-BM	APS, Sector 22-ID	APS, Sector 22-ID	APS, Sector 22-BM	APS, Sector 22-BM	APS, Sector 22-BM	APS, Sector 22-BM
space group	$P2_22_12_1$	$P2_22_12_1$	$P2_22_12_1$	$P2_22_12_1$	$P2_22_12_1$	$P2_22_12_1$	$P2_22_12_1$	$P2_22_12_1$
unit cell lengths (Å)	a=88.27, b=141.89, c=172.92	a=88.58, b=142.00, c=174.38	a=88.40, b=142.12, c=174.41	a=88.57, b=142.72, c=175.01	a=88.36, b=141.75, c=174.37	a=88.53, b=141.98, c=173.80	a=88.57, b=141.56, c=174.63	a=88.33, b=141.35, c=173.53
unit cell angles (˚)	α=β=γ=90°	α=β=γ=90°	α=β=γ=90°	α=β=γ=90°	α=β=γ=90°	α=β=γ=90°	α=β=γ=90°	α=β=γ=90°
wavelength (Å)	0.8	1.0	0.8	0.8	1.0	1.0	1.0	1.0
temperature (K)	100	100	100	100	100	100	100	100
resolution (Å) [a]	45.00-2.20 (2.24-2.20)	35.00-2.00 (2.07-2.00)	35.00-1.98 (2.03-1.98)	45.00-2.15 (2.19-2.15)	45.00-1.95 (1.98-1.95)	50.00-2.00 (2.03-2.00)	50.00-2.15 (2.15-2.19)	50.00-2.20 (2.24-2.20)
completeness (%)[a]	99.8 (99.4)	99.8 (98.9)	95.2 (99.7)	99.9 (100.0)	94.9 (88.8)	97.2 (97.4)	99.9 (100)	99.6 (99.2)
R_{merge} (%)[a, b]	8.0 (53.5)	11.2 (89.1)	10.7 (78.3)	12.2 (84.1)	10.4 (84.3)	10.8 (74.5)	14.8 (58.7)	9.9 (71.7)
$I/\sigma I$ [a]	45.2 (4.4)	27.3 (2.3)	22.7 (2.2)	27.1 (2.7)	28.4 (2.2)	13.3 (3.2)	17.7 (3.9)	36.9 (3.7)
multiplicity [a]	14.0 (11.6)	13.7 (9.1)	9.8(7.8)	12.5 (10.3)	10.5 (6.4)	11.9 (10.5)	8.8 (7.6)	11.4 (10.3)
no. of observed reflections	109724	149605	149047	120432	152910	146528	119893	110554
**Refinement**								
resolution (Å)	2.20	2.00	2.00	2.15	1.95	2.00	2.15	2.20
no. reflections; working/test	109619/5469	149298/7485	147418/7401	114123/6029	145080/7649	144988/7303	119216/5973	110312/5518
R_{work} (%)[c]	18.6	17.2	16.2	18.0	17.6	19.5	19.2	18.4
R_{free} (%)[d]	23.9	21.8	20.8	23.7	21.6	23.7	23.5	22.7

no. of protein atoms	14651	14684	14684	14684	14684	14668	14668	14668
no. of ligand atoms	23	188	220	220	220	204	220	220
no. of solvent sites	702	1682	1397	802	1702	1188	1136	882
**Average B-factor (Å^2)**								
protein	41.1	28.5	28.5	39.7	27.9	31.7	29.2	36.2
NAD^+	N/A	28.8	40.1	56.7	37.5	37.0	41.4	56.4
NA^+	35.3	N/A	32.3	41.6	30.7	34.0	43.6	43.9
EG/GOL/AMS/HMS	38.4	42.7	32.4	45.6	23.0	42.1	25.1	28.1
solvent	42.7	37.3	37.5	43.3	35.4	37.4	34.9	39.2
**Ramachandran statistics[e]**								
preferred (%)	95.36	95.58	95.74	94.54	96.10	95.32	95.53	95.69
allowed (%)	4.22	4.05	3.85	4.89	3.59	4.31	4.21	3.79
**Root mean square deviation**								
bond lengths (Å)	0.014	0.012	0.019	0.017	0.009	0.009	0.008	0.009
bond angles (˚)	1.369	1.426	1.810	1.798	1.189	1.194	1.209	1.183
PDB code	4I26	4I1W	4I25	4I2R	4NPI	4OE2	4OU2	4OUB

[a] Values in parentheses are for the highest resolution shell.

[b] Rmerge = $\Sigma i\ |I_{hkl,i} - \langle I_{hkl}\rangle| / \Sigma_{hkl}\ \Sigma i\ I_{hkl,i}$, where $I_{hkl,i}$ is the observed intensity and $\langle I_{hkl}\rangle$ is the average intensity of multiple measurements.

[c] Rwork = $\Sigma||F_o|-|F_c||/\Sigma|F_o|$, where $|F_o|$ is the observed structure factor amplitude, and $|F_c|$ is the calculated structure factor amplitude.

[d] R_{free} is the R factor based on 5% of the data excluded from refinement.

[e] Based on values attained from refinement validation options in COOT

Supplementary Table 2. In order to assess the degree of out-of-plane distortion, the angles from the plane of the carbon backbone of the substrate (2-HMS) to the oxygen at the C6 position of the substrate-enzyme adducts was measured in degrees. The intermediate assigned as a thiohemiacetal adduct shows significantly more out-of-plane bending than the thioacyl intermediates.

	wt-thioacyl	E268A-thioacyl	E268A-thiohemiacetal
PDB entry	4NPI	4OUB	4OU2
Subunit A	23.5	18.1	44.4
Subunit B	28.7	22.3	51.9
Subunit C	22.0	26.5	63.5
Subunit D	30.5	14.9	60.3
Average ± SD	26.2 ± 4.1	20.5 ± 5.1	55.0 ± 8.6

Supplementary Table 3. Relative energy of 2-AMS optimized in the imine tautomer at the B3LYP/6-31G*+ level of theory with the presence of Arg120 and Arg464 guanidinium groups.

	Dihedral Angle[a] (degrees)	Relative Energy[b] (kcal mol^{-1})
'***Z***-like'	0	0.18
	45	2.23
	90	8.47
	135	4.06
'***E***-like'	180	0.00

[a] The angle about the 2-3 bond of 2-AMS, see Fig. 6b in the main text, was constrained.

[b] The lowest energy structure ('***E***-like') was set as zero.

Supplementary Discussion

Secondary Structure Features. Each polypeptide contains 500 amino acids, but the first 16 - 17 amino acids are not included in our model due to missing electron density even though full-length AMSDH was used for crystallization. It is most likely that these N-terminal residues belong to a random coil as in most other ALDH structures. The first 135 residues comprise a cap which surrounds the cofactor binding domain. This cap region starts with two β-hairpin motifs (residues 22 - 50) and is followed by four α-helixes (residues 51 - 134). The sequence then extends to the subunit interaction domain with two beta strands (residues 138 -158). The central strand of the cofactor binding domain starts at residue 161 and stops at residue 266 and resembles a distorted Rossmann fold. The catalytic domain (residues 267 - 476) is based on a topologically related βαβ polypeptide fold and contains a thiol, Cys302, in the catalytic center (**Supplementary Figure 1**). The sequence ends with a C-terminal helix and a beta strand (residue 477 - 500), which is part of the oligomerization domain. The active site is located in the region between the NAD^+ binding domain and the catalytic domain with entrances for NAD^+ and primary substrate on two separate sides.

Binary and Ternary Complex Features. The adenine ribose ring and the two phosphate groups are the main components that stabilize the NAD^+ position by interacting with protein residues which all belong to the surrounding loops, except Thr250, which belongs to the 8th α-helix. The nicotinamide half of NAD^+ has less interaction with local residues. Two oxygen atoms (O2 and O3) of the ribose ring form hydrogen bonds with OE2 and OE3 of Glu404, respectively. On the other ribose ring, the O3 atom forms an H-bond with the Nz of Lys192 while its O2 atom forms a hydrogen bonds with both Lys192 (Nz) and Glu195 (OE1). The O1 belonging to the phosphate

group nearer the nicotinamide is hydrogen-bonded to NE1 of Trp168. The anionic O2 of the other phosphate group forms hydrogen bonds with both Thr250 (OG1) and Glu247 (N) (**Fig. 2e**).

For the 2-AMS and 2-HMS binding pocket, except for the hydrogen bonds provided by Arg120 and 464, the substrate binding pocket is mostly composed of hydrophobic residues including four leucine residues (170, 173, 174, and 303), one valine (Val301), and one phenylalanine residue (Phe470) which rotates upon substrate binding, and they are all conserved residues (**Supplementary Figure 9**). This hydrophobic residue cluster may also help stabilize the loop on which Arg464 is located through hydrophobic interactions with Tyr462 and Trp461.

Supplementary Methods

Expression and Purification of *pf*AMSDH and *pf*ACMSD. To construct a His_{10}-tagged AMSDH expression plasmid, *nbaE* gene from *P. fluorescens* (accession: AB088043.2) encoding AMSDH was amplified by the polymerase chain reaction (PCR) using genomic DNA of *P. fluorescens* strain KU-7 as a template and primers 5'-GGAATTCCATATGAATACCTTACCAAGTCAAG-3' and 5'-CCCTCGAGTTAAATTTTTATGCAGATGTTGG-3' (built-in *Nde*I and *Xho*I sites are underlined). The PCR product was purified from a 0.8% agarose gel, digested with *Nde*I and *Xho*I, and ligated in the equivalent sites of pET-16b (Novagen). Ligation product was transformed to *Escherichia coli* BL21(DE3) for protein expression. A single colony was introduced to 10 mL of autoclaved LB medium containing 100 μg/mL ampicillin and cultured at

37 °C. When cells reached ca. 0.6 OD at 600 nm, 1.5 mL of cells were diluted into 500 mL autoclaved LB medium containing ampicillin. The cells were cultured in 37 °C until the optical density reached ca. 0.8 at 600 nm. Isopropyl β-D-1-thiogalactopyranoside was then added to a final concentration of 0.6 mM, and the temperature was lowered to 28 °C for 12 hours to induce AMSDH expression before the cells were harvested by centrifugation at 8,000 g. The harvested cells were then resuspended in 50 mM potassium phosphate buffer, pH 8.0, containing 300 mM NaCl and 5% glycerol. The cell slurry was passed through an M-110P Microfluidics cell disruptor and the debris was removed by centrifugation at 27,000 g for 30 min at 4°C. The supernatant containing AMSDH was purified using a Ni-NTA affinity column on an ÄKTA FPLC system (GE Healthcare). The major fraction with AMSDH activity was eluted by increased imidazole concentration. Purified protein was concentrated and desalted on a pre-packed HiTrap desalting column (GE Healthcare) using buffer containing 50 mM HEPES (pH 7.5), 150 mM NaCl, and 1 mM DTT. Expression, purification and protein re-constitution of ACMSD were performed as described previously[1].

Site-directed Mutagenesis. C302S, E268A, R120A, and R464A single mutation variants were constructed by the PCR overlap extension mutagenesis technique[2]. Plasmid containing AMSDH from *P. fluorescens* was used as a template. The forward primers used in the site-directed mutagenesis are 5'-CAACTCGGGGCAGGTCagcCTGTGTTCCGAACG-3' for C302S, 5'-GAAAGAAGTGTCTTTCgcgTTGGGGGGCAAGAACG-3' for E268A, 5'-GGACCCTCGATATTCCTgcgGCCATTGCCAACTTTC-3' for R120A, and 5'-GAACACCTGGTACTTGgcgGATCTGCGTACGCC-3' for R464A. The insert of each mutant was verified by DNA sequencing and the positive clone was transformed to E. coli BL21(DE3). The expression and purification of the mutants are the same as wtAMSDH.

Supplementary References

1. Huo, L., Davis, I., Chen, L. & Liu, A. The power of two: arginine 51 and arginine 239* from a neighboring subunit are essential for catalysis in α-amino-β-carboxymuconate-ε-semialdehyde decarboxylase. *J. Biol. Chem.* **288**, 30862-30871 (2013).
2. Horton, R.M. PCR-mediated recombination and mutagenesis. SOEing together tailor-made genes. *Mol. Biotechnol.* **3**, 93-9 (1995).
3. Gouet, P., Robert, X. & Courcelle, E. ESPript/ENDscript: Extracting and rendering sequence and 3D information from atomic structures of proteins. *Nucleic Acids Res.* **31**, 3320-3 (2003).
